# Identification of receptor-like proteins induced by *Sclerotinia sclerotiorum* in *Brassica napus*

**DOI:** 10.3389/fpls.2022.944763

**Published:** 2022-08-16

**Authors:** Wei Li, Junxing Lu, Chenghuizi Yang, Shitou Xia

**Affiliations:** ^1^Hunan Provincial Key Laboratory of Phytohormones and Growth Development, College of Bioscience and Biotechnology, Hunan Agricultural University, Changsha, China; ^2^College of Life Science, Chongqing Normal University, Chongqing, China

**Keywords:** *Brassica napus*, receptor-like protein, evolution, expression pattern, *Sclerotinia sclerotiorum*

## Abstract

Heightening the resistance of plants to microbial infection is a widely concerned issue, especially for economical crops. Receptor-like proteins (RLPs), typically with tandem leucine-rich repeats (LRRs) domain, play a crucial role in mediating immune activation, being an indispensable constituent in the first layer of defense. Based on an analysis of orthologs among *Brassica rapa, Brassica oleracea*, and *Brassica napus* using *Arabidopsis thaliana* RLPs as a reference framework, we found that compared to *A. thaliana*, there were some obvious evolutionary diversities of RLPs among the three *Brassicaceae* species. *BnRLP* encoding genes were unevenly distributed on chromosomes, mainly on chrA01, chrA04, chrC03, chrC04, and chrC06. The orthologs of five *At*RLPs (*At*RLP3, *At*RLP10, *At*RLP17, *At*RLP44, and *At*RLP51) were highly conserved, but retrenchment and functional centralization occurred in *Brassicaceae* RLPs during evolution. The RLP proteins were clustered into 13 subgroups. Ten *BnRLPs* presented expression specificity between R and S when elicited by *Sclerotinia sclerotiorum*, which might be fabulous candidates for *S. sclerotiorum* resistance research.

## Introduction

Integrating exterior information with intrinsic cues is indispensable for all organisms, which is particularly crucial for plants due to their sessile lifestyle. Plants employ a large number and a vast variety of receptors for transducing signals from the extracellular matrix to the cell interior. Receptor-like proteins (RLPs), known as a kind of pattern recognition receptors (PRRs), are the main components of the first layer of plant immunity and a vital group of transmembrane receptors, which are generally composed of a short cytoplasmic domain, an extracellular leucine-rich repeat (LRR) domain, and a transmembrane domain (Wang et al., 2008). To date, 57 *At*RLPs (Wang et al., 2008), 90 RLPs in rice (Fritz-Laylin et al., 2005), 82 RLPs in poplar (Petre et al., 2014), 144 RLPs in cotton (Chen et al., 2015), 176 RLPs in tomato (Kang and Yeom, 2018), and 228 RLPs in *Brassica juncea* (Yang et al., 2020) have been identified, and pan-genome prediction of resistance gene analogs (RGAs) in *Brassica oleracea* and also across the *Brassicaceae* were conducted (Bayer et al., 2019; Tirnaz et al., 2020). Currently, the enormous variation in RLPs between or within species has been discussed. RLPs are much more diverse and function in various pathways, involving both plant developmental regulation and immune response, even possessing dual roles. Indeed, most RLPs with assigned functions are involved in disease resistance. The identification of tomato Cf and Ve proteins that provide resistance against *Cladosporium fulvum* and *Verticillium spp*., respectively, started an era of RLP research (Jones et al., 1994; Kawchuk et al., 2001).

Subsequently, RLPs, for example, apple HcrVfs that confer resistance to fungal pathogens *Cladosporium fulvum* as well as *Venturia inaequalis* (Belfanti et al., 2004; Malnoy et al., 2008); tomato LeEIX proteins that function as a receptor to mediate recognition of the ethylene-inducing xylanase of *Trichoderma viride* (Ron and Avni, 2004); *Nicotiana benthamiana* elicitor-inducible leucine-rich repeat receptor-like protein (EILP) that is an ortholog of tomato Cf protein involved in bamboo mosaic virus movement (Chen et al., 2017); and Gbvdr6 that was homologous to Ve conferring resistance to *Verticillium dahliae Kleb* through the regulation of the JA/ET and SA signaling pathways in upland cotton were identified successively (Yang et al., 2017).

In *Arabidopsis*, of the 57 putative RLPs, approximately a quarter of them have been functionally characterized. CLAVATA2 (CLV2)/*At*RLP10 and TOO MANY MOUTHS (TMM)/*At*RLP17 are the first two *At*RLPs that are experimentally validated implicating in the development functions and are involved in regulating the meristem and organ development (Jeong et al., 1999), as well as stomatal patterning and distribution (Nadeau and Sack, 2002), respectively. FASCIATED EAR2, an ortholog of CLV2 in maize, also plays an important role in the regulation of stem cell homeostasis (Taguchi-Shiobara et al., 2001). It is noteworthy that functional redundancy exists among *At*RLPs. *At*RLP2 and *At*RLP12, identified as homologs of CLV2, rescue the *clv2* meristem defects when driven by the endogenous promoter of *CLV2* (Wang et al., 2010). Besides, the overexpression of *AtRLP3* and *AtRLP11* also rescued the phenotype of the *clv2-1* mutant (Wu et al., 2016a). Nevertheless, Steidele and Stam clustered *At*RLPs into two superclades (basal RLPs and pathogen-responsive RLPs) and referred to nine *At*RLPs (*At*RLP4, 10/CLV2, 17/TMM, 29, 44, 46, 51, 55, and 57) as putative developmental orthologs (PDOs) based on differences in transcript and protein abundance or clustering at the genomic loci (Steidele and Stam, 2021). Interestingly, CLV2/*At*RLP10 plays a role both in developmental and defense-related processes.

Over the years, more *At*RLPs have been shown to preferentially fulfill a role in pathogen defense. Among them, six *At*RLPs (RLP1, 3, 23, 30, 32, and 42) were validated as pathogen-responsive RLPs (Steidele and Stam, 2021). *At*RLP1/ReMAX is required for the perception of eMAX, which is a kind of MAMPs from *Xanthomonas* (Jehle et al., 2013a,b). *At*RLP3/RFO implicates in resistance to the vascular wilt fungus *Fusarium oxysporum* (Shen and Diener, 2013). *At*RLP23 perceives a conserved fragment found in most necrosis and ethylene-inducing peptide1-like proteins (NLPs) and thereby activates the immune responses, which are involved in pre-invasive resistance to the pathogen *Botrytis cinerea* (Albert et al., 2015; Ono et al., 2020). *At*RLP30 and *At*RLP18 impact the susceptibility of *Pseudomonas syrinegae* pv. *phaseolicola* to non-host resistance (Wang et al., 2008). Additionally, *At*RLP30 involves resistance toward necrotrophic fungal pathogen *Sclerotinia sclerotiorum* as well (Zhang et al., 2013). *At*RLP32 is confirmed as the receptor of proteobacterial translation initiation factor 1 (IF1) (Fan et al., 2022). *At*RLP42/RBPG1 recognizes endopolygalacturonases from the plant pathogen *Botrytis cinerea* and saprotroph *Aspergillus niger* (Zhang et al., 2014). *At*RLP44 activates brassinosteroid signaling through interaction with BAK1 (Wolf et al., 2014). *At*RLP51/SNC2 and *At*RLP55 implicate in basal defense against the bacterial pathogen *Pseudomonas syringae* pv *tomato* DC3000 (Zhang et al., 2010). *At*RLP52 is suggested to influence resistance toward the powdery mildew pathogen *Erysiphe cichoracearum* (Ramonell et al., 2005). Previous studies indicated that disease resistance genes are more likely to be duplicated and underwent functional divergence compared to growth-related genes, and the most homologous *AtRLP* genes anchored at the same or adjacent locus and underwent massive duplications (Fritz-Laylin et al., 2005; Wang et al., 2008; Wu et al., 2016a). Generally, RLPs need to constitutively interact with additional components, such as RLKs, to activate cellular responses. For example, SUPPRESSOR OF BAK1-INTERACTING RECEPTOR-LIKE KINASE 1 (SOBIR1) is required for RLP-mediated resistance to bacterial, fungal, and oomycete pathogens. SOBIR1 physically interacts with Cf-4, Ve1, *At*RLP1, *At*RLP23, *At*RLP30, *At*RLP32, and *At*RLP42, indicating the existence of crosstalk among different RLP signaling pathways (Wang et al., 2008; Jehle et al., 2013a; Liebrand et al., 2013; Zhang et al., 2013, 2014; Albert et al., 2015; Ma and Borhan, 2015; Catanzariti et al., 2017). Apart from the involvement in biotic stress responses, RLP was also engaged in abiotic stress tolerance. Currently, *At*RLP28 is the only one found to play a role in salt stress tolerance (Wu et al., 2016a).

*Brassica napus* is one of the most economically important oil crops in the world. Its yield and quality, however, are affected remarkably by multiple external stimuli, especially the invasion of major *Brassica* pathogenic fungi, such as *S. sclerotiorum* and *Leptosphaeria maculans. Brassica napus* (AACC) are an allotetraploid generated by recombination of two diploid genomes, *Brassica rapa* (AA) and *B. oleracea* (CC). *Brassica napus* and its progenitor species share significant homology with *Arabidopsis thaliana* (Nagaharu, 1935). Currently, several pathogen-responsive RLPs were identified across *Brassica* species. Among them, *LepR3* and *Rlm2*, which co-localized within the same genetic interval of blackleg resistance locus, were cloned from *B. napus* and rendered race-specific resistance to fungal pathogen *L. maculans* upon recognition of different cognate Avr proteins, AvrLm1 and AvrLm2, respectively, and both of them require SOBIR1 for their functions (Larkan et al., 2013, 2015; Ma and Borhan, 2015). Another two *RLP* genes identified from *B. rapa* are *Bra032747* and *Bra032746*, which confer resistance to *Brassica* downy mildew (Zhang et al., 2018). Pathogen-associated cell-surface receptors are important for perceiving immunogenic signals in the challenged host (Kourelis and van der Hoorn, 2018). However, only a limited batch of *RLPs* was functionally characterized in *Brassica* species. Here, we performed the identification and phylogenetic analysis of *RLP*s in *B. napus* and its progenitors, *B. rapa* and *B*. *oleracea*, which are highly homologous to *At*RLPs, and sorted out their distributions on chromosomes. To further assume the possible functions of identified *RLPs*, we investigated gene expression profiles of *B. napus* elicited by *S. sclerotiorum* utilizing the published pathogen-induced RNA-seq datasets. Taken together, these results will sketch out the basic information across identified *Cruciferae RLPs*, contributing to deciphering genome evolution and duplication, as well as elucidation of *RLP* gene function in detail.

## Materials and methods

### Identification of *RLP* genes

*Arabidopsis thaliana* genomic and annotation data were downloaded from the TAIR (http://www.arabidopsis.org). Genomic and annotation data of *B. napus* and *B. rapa* were downloaded from BRAD (http://brassicadb.cn/; Chen et al., 2022). *Brassica oleracea* genomic and annotation data were downloaded from Ensemblplants (https://plants.ensembl.org/index.html).

In *A. thaliana*, a typical RLP protein consists of tandem LRR motifs, along with SP (signal peptide) and TM (transmembrane) in two terminals (Wang et al., 2008). But these LRR motifs vary in number and distribution, thus they are not competent as a good query for protein screening. Given this, two methods were used for the identification of RLP-encoding genes in this work. First, as queries, 55 identified *Arabidopsis* RLP protein sequences (two pseudogenes, *AtRLP18* and *AtRLP49*, excluded in this work) were obtained and used to search analogs in three *Brassica* species using the BLASTP program based on a comparative genomics approach, thereby obtaining the first part of the candidate RLP proteins. Furthermore, RLP-encoding genes were also screened through Hidden Markov Model (HMM) profiles built by the model corresponding to 55 *At*RLP proteins using the HMMSearch program (https://www.ebi.ac.uk/Tools/hmmer/). Subsequently, the two sets of candidates were merged and subjected to NCBI-BLASTP using UniProtKB/SwissoPrpt, and their annotations were downloaded for further screening.

### Gene structure, conserved motif, and phylogenetic analyses

The exon–intron organization of the RLP-encoding genes was depicted using GSDS2.0 (Hu et al., 2015), and the conserved motifs in RLPs were searched using the MEME suite (Bailey and Elkan, 1994). The SP and TM were predicted using SignalP-5.0 Server (http://www.cbs.dtu.dk/services/SignalP/), SMART (http://smart.embl-heidelberg.de/), TMHMM Server v. 2.0 (http://www.cbs.dtu.dk/services/TMHMM/), and Pfam (http://pfam.xfam.org/). Approximately 2,000-bp upstream flanking fragments of the RLP genes were derived from the genome and used for promoter cis-element prediction using PlantCARE (Lescot et al., 2002). The visualization was completed using TBtools (Chen et al., 2020). The phylogenetic analyses of the selected *B. napus* and *Arabidopsis* RLP proteins were generated using MEGA7.0 with the maximum likelihood (ML) algorithm. Bootstrap analysis with 1,000 replications was performed to assess group support.

### Physical location of *RLP*s on the chromosome

The chromosome location of *Brassicaceae RLP*s was obtained from the genome annotation files. The distribution and tandem duplication of *Brassicaceae RLP* genes on chromosomes was generated and depicted using TBtools (Chen et al., 2020).

### Expression characteristics of *BnRLP* genes induced by pathogens

The RNA-seq data were obtained from NCBI. Data calibrations were performed, and some data with big deviations were removed. A heat map was drawn by HemI 1.0.3.3 (Deng et al., 2014). A mid-resistant *B.napus* variety Zhongyou821 was used for *S. sclerotiorum* inoculation and assessment of *BnRLP* gene expression. Plants were grown in a greenhouse with artificial irrigation. At the flowering stage, stems and leaves were inoculated with mycelia on living plants, harvested after 48 h inoculation, flash-frozen in liquid nitrogen, and stored at −80°C. Three plants with the closest phenotype and growth status were harvested, and harvesting was repeated three independent times. qRT-PCR was performed as described above with slight modifications (Li et al., 2016). Total RNA samples were isolated from rapeseed tissues using the Plant RNAprep Pure Kit (Tiangen, Beijing). RNA was quantified on a NanoDrop 1000 (NanoDrop Technologies, Inc.), and RNA integrity was evaluated on a 1% agarose gel. RNA was transcribed into cDNA using a GoScript RT Reagent Kit (Promega, USA). Primers used for qRT-PCR were designed using the Primer Premier 5.0 program to target the ORF of each gene with an amplicon sized between 80 and 200 bp (Supplementary Table 3). UBC9 served as a reference gene (Chen et al., 2010). qRT-PCR was performed using 10-fold diluted cDNA and a Universal SYBR Green Supermix Kit (Bio-RAD, USA) on a CFX96 real-time PCR machine (Bio-Rad, USA) according to the MIQE Guidelines (Minimum Information for Publication of Quantitative Real-Time PCR Experiments; Bustin et al., 2009).

## Results

### Identification and characterization of RLP-encoding genes in *B*. *napus*

Since *A. thaliana* is a close relative of *B. napus* (rapeseed), 55 *At*RLP protein sequences (two pseudogenes, *At*RLP18 and *At*RLP49, excluded in this work) were downloaded from the *Arabidopsis* database (TAIR) and used as queries to identify the candidate RLP proteins in rapeseed by BLAST search against BRAD or Ensemblplants database. In addition, Hidden Markov Model (HMM) was used for further confirmation of candidate RLPs. To better understand the evolution of RLPs, *B*. *rapa* and *B*. *oleracea* genomes were also selected for the screening of orthologs. In total, 118, 173, and 276 RLPs were identified from *B. rapa* (AA), *B. oleracea* (CC), and *B. napus* (AACC), respectively (Supplementary Table 1). Although a pangenome investigation of resistance gene analogs (RGAs) across some *B. oleracea* varieties had identified 213 candidates (Bayer et al., 2019), we conducted a further fine selection to get a more reliable but attenuated set of BoRLPs. Then, all *RLP* CDSs were downloaded and aligned to corresponding genomic DNA to obtain high-confidence sequences. Sequence alignment revealed a great diversity of *BnRLP*s (Supplementary Figure 1). The number of introns ranged from 0 to 22 (*BnaC06g23240D*), and ~16% *BnRLP*s had no introns which were sharply attenuated compared to that of *AtRLP*s (70%). The orthologs of five *AtRLP*s (*AtRLP3, AtRLP10, AtRLP17, AtRLP44*, and *AtRLP51*) did not evolve any intron, showing high conservation. Apart from quantitative variation of *BnRLP* introns, some *BnRLP* genes possessed extra-long introns (more than 2,000 bp), like *BnaA01g31040D* (intron I, 5,336 bp), *BnaA09g35430D* (intron II, 4,756 bp; intron III, 5,018,bp), *BnaC02g22740D* (intron III, 6,474bp), and *BnaC02g22760D* (intron X, 5,030 bp). *AtRLP9* (*AT*1G58190.1) also contained long introns (3,437 bp), but it had no corresponding orthologs in *Brassicaceae* species. Rapeseed amplified the RLP*-*encoding gene copies by a wide margin, and the tendency of increasing intron number and length variation was available for alternative splicing and further benefit for functional diversity.

**Figure 1:**
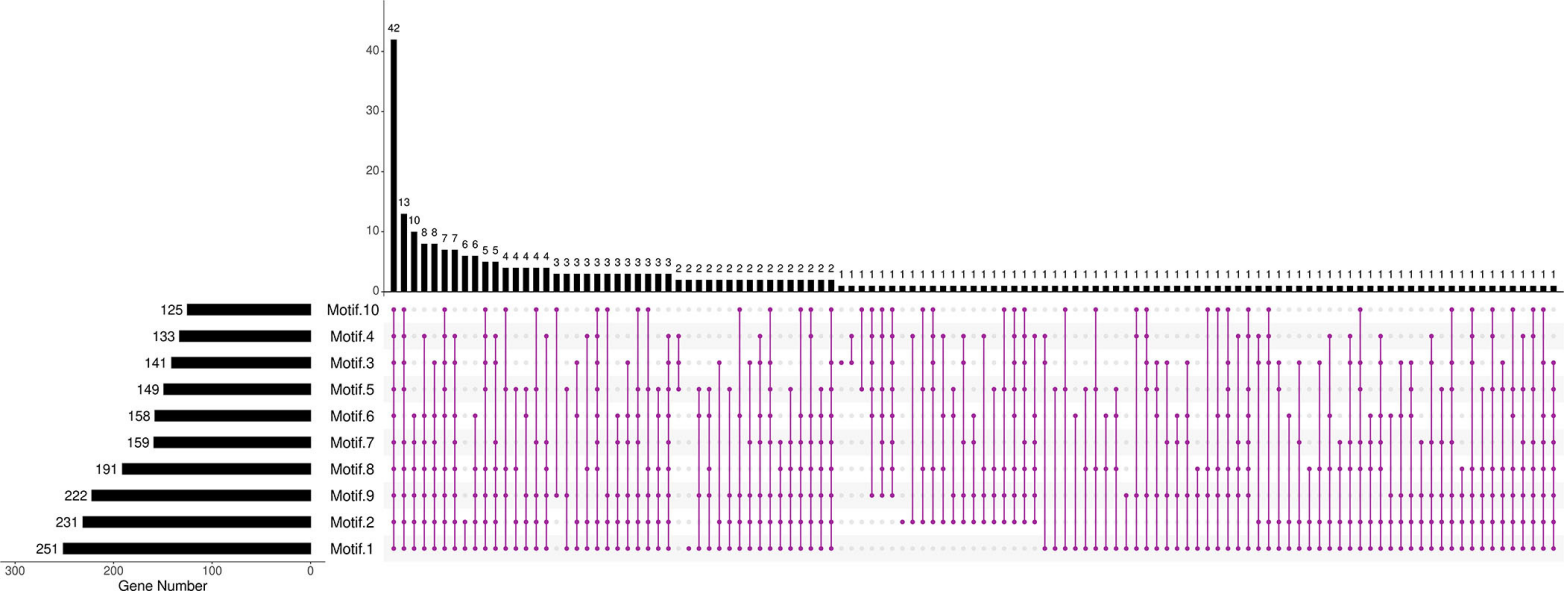
Distributions of motifs in *Bn*RLPs. The Upset plot shows the distribution of motifs in the *Bn*RLPs. The number chart above represents the number of genes contained in each type of RLP. The bar chart at the bottom left represents the number of events included in each type of motif. The dotted line shows the types of motifs contained in the group.

Further analysis revealed that the composition and the number of conserved motifs or domains varied to a large extent. The length of amino acids ranged from 73 (*Bna*Cnng19280D) to 2,736 aa (*Bna*C06g23240D; Supplementary Figure 2). Ten motifs were predicted in *Bn*RLP proteins (Figure 1, Supplementary Figure 3). Motif 1, Motif 2, and Motif 9 were distributed on more than 80% *Bn*RLP proteins, and the average number on each protein was 2.3, 1.7, and 2.2, respectively. More than 15% of *Bn*RLP proteins possessed all 10 motifs, and 42 of the 276 had less than five types. Only a quarter of *Bn*RLP proteins met the characteristics of canonical RLP protein, namely composed of the SP and TM domains at the ends and the tandem LRR domains in the middle (Supplementary Figure 1).

### Chromosomal localization and gene expansion

**Figure 2:**
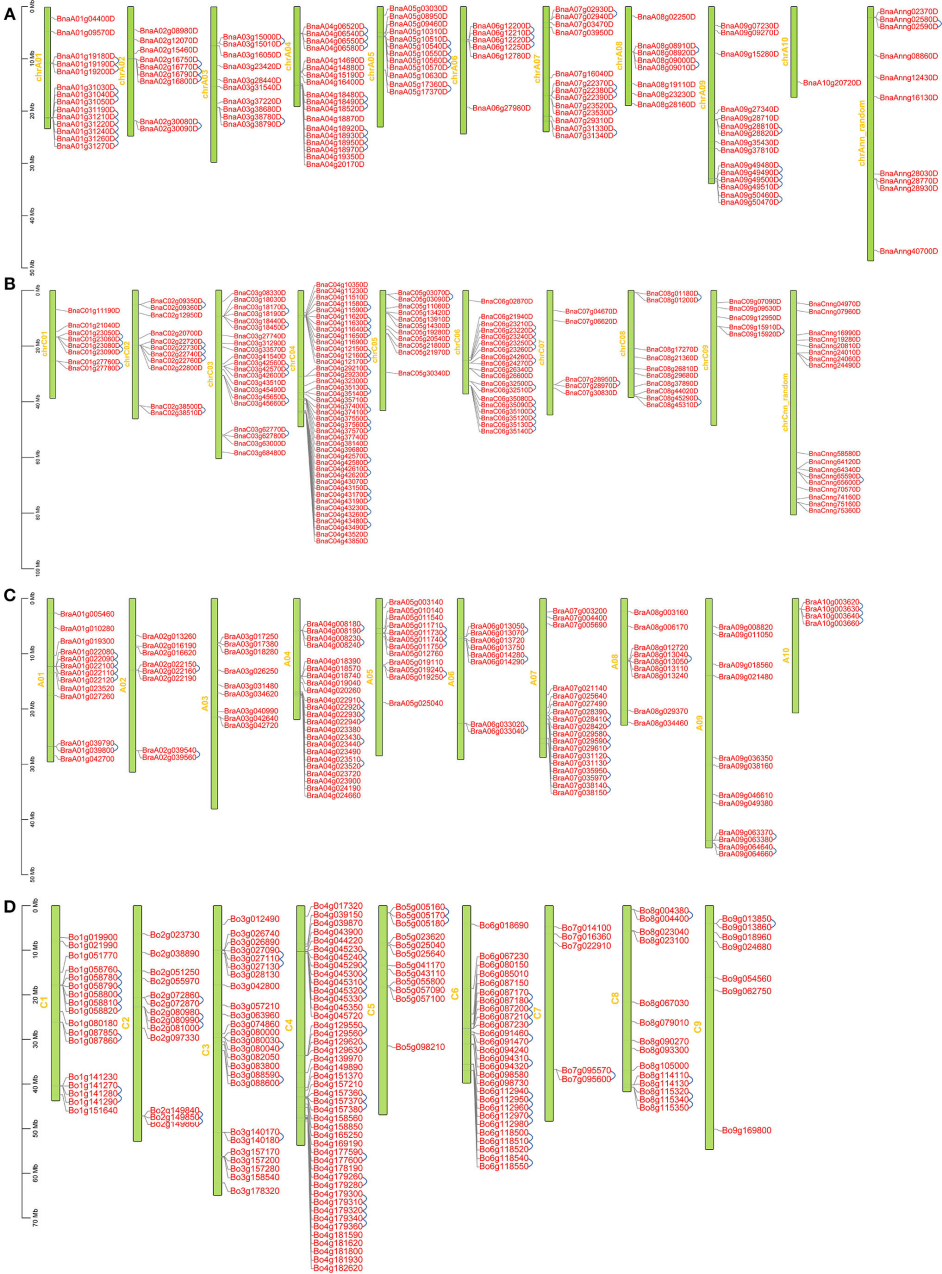
Locations of *RLP* genes on chromosomes in *Brassica napus, Brassica rapa* and *Brassica oleracea*. **(A, B)** Locations of *RLP* genes on chromosomes in *B. napus*; **(C)** Locations of *RLP* genes on chromosomes in *B. rapa*. **(D)** Locations of *RLP* genes on chromosomes in *B. oleracea*. A01-A09, C01-C08, and chrA01-chrC09 represent the chromosome number in *B. rapa, B. oleracea*, and *B. napus*, respectively. The blue line represents tandem duplication.

*BnRLP* encoding genes were unevenly distributed on eighteen chromosomes (Figure 2). The 116 and 148 *BnRLP*s were mapped onto chrA (Figure 2A) and chrC (Figure 2B) subgenome, respectively, and many of these genes reside in a cluster manner. The percentage of *RLP* genes on chromosomes in clusters in *B. oleracea* (51.22%) and *B. rapa* (49.14%) was lower than that of *B. napus* (56.06%). The numbers of genes in clusters ranged from two to six in *B. napus*, and the maximum gene number in *B. oleracea* and *B. rapa* were 7 and 5, respectively. In *B. napus*, 148 RLP genes were located in 55 clusters and the remaining 221 genes were singletons. Among these clusters, 12 with 29 genes were located on chrC04 (Figure 2B), which was similar to that in *B. oleracea* (seven clusters containing 23 genes on chromosome C04) (Figure 2D). The *B. rapa* genome carries 57 RLP genes in 23 clusters (Figure 2C). Compared to that in *B.napus*, the same number of clusters but fewer RLP genes (57 and 67 in *B. rapa* genome and *B.napus* A-subgenome) were observed in *B. rapa*. The cluster distribution between the two species was obviously different on chromosome A01 and chrA01, showing that *B.napus* evolved more tandem *RLP* genes on chrA01. Chromosome chrC04 contained the largest number of *Bn*RLP genes (41 genes), followed by chrC03 (21 genes), chrC06 (19 genes), chrA04 (18 genes), and chrA01 (14 genes). Only one *BnRLP* gene was located on chrA10. *RLP* genes were assigned tandemly on most chromosomes, especially on chrA01, chrA04, chrA05, chrA07, chrC04, and chrC06, which presented higher distribution densities. Generally, the distribution of *BnRLP* genes on the A or C subgenome was similar to that of *BrRLP* or *BoRLP* genes in their respective genomes.

**Figure 3:**
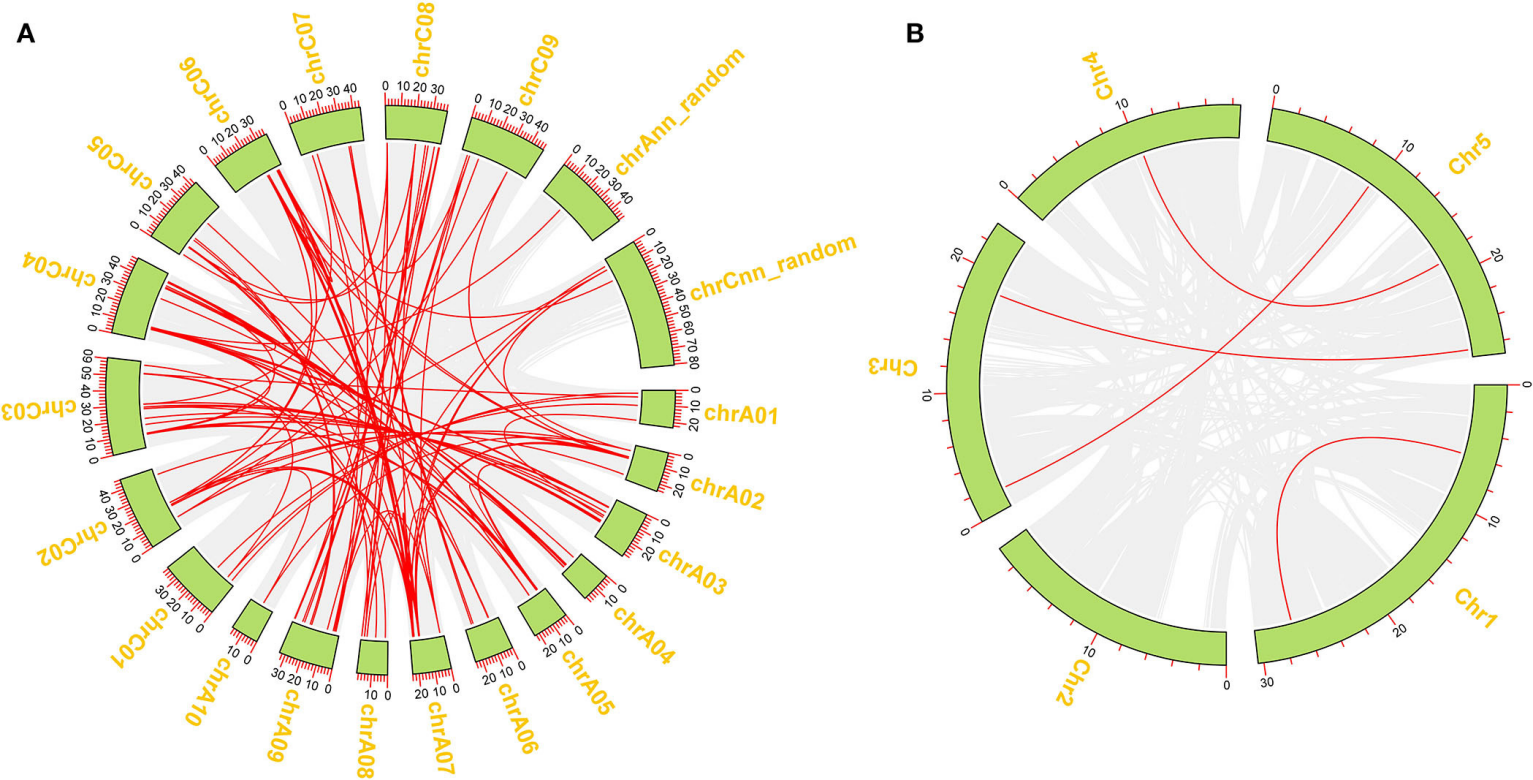
Duplication of *BnRLP* and *AtRLP* genes. **(A)** Duplication of *RLP* genes in *Brassica napus*. **(B)** Duplication of *RLP* genes in *Arabidopsis thaliana*. The red line represents the gene pairs and the green box represents the genome.

To better reveal the expansion of *RLP* genes in the rapeseed genome, the duplication patterns of 276 *BnRLP* genes were predicted and analyzed by MCScanX (Wang et al., 2012) and visualized by TBtools (Chen et al., 2020), and the result showed that rapeseed had 36 times as many *RLP* gene pairs as *Arabidopsis* (Figure 3). Moreover, the possible syntenic relationship of RLP-encoding genes between *A. thaliana* and *Brassica* genomes was also investigated. Subsequently, we obtained 59 orthologous gene pairs between *A*. *thaliana* and *B*. *oleracea*, 50 between *A*. *thaliana* and *B*. *rapa*, 250 between *B*. *oleracea* and *B*. *napus*, and 223 between *B*. *rapa* and *B*. *napus*, which are shown in Supplementary Figure 4. Ka/Ks analysis was performed by TBtools (Chen et al., 2020) between *A. thaliana* and the other three *Brassica* species(*B. napus, B. rapa* and *B. oleracea*), respectively. The Ka/Ks value of all pairs was <1 (Supplementary Table 2), suggesting that the main force for the evolution of those RLP gene pairs was negative selection. Most orthologous *AtRLP* genes are located on chromosomes 1 and 2, and correspondingly, chromosomes A04/A07 and C04/C06 possessed the higher orthologous regions in *B. rapa* and *B. oleracea*, respectively. Approximately, the aliquot of orthologous *BnRLP* genes was resourced from *B. rapa* and *B. oleracea* and numerically distributed evenly on chrA and chrC subgenomes.

### Evolution analysis of *Bn*RLPs

**Figure 4:**
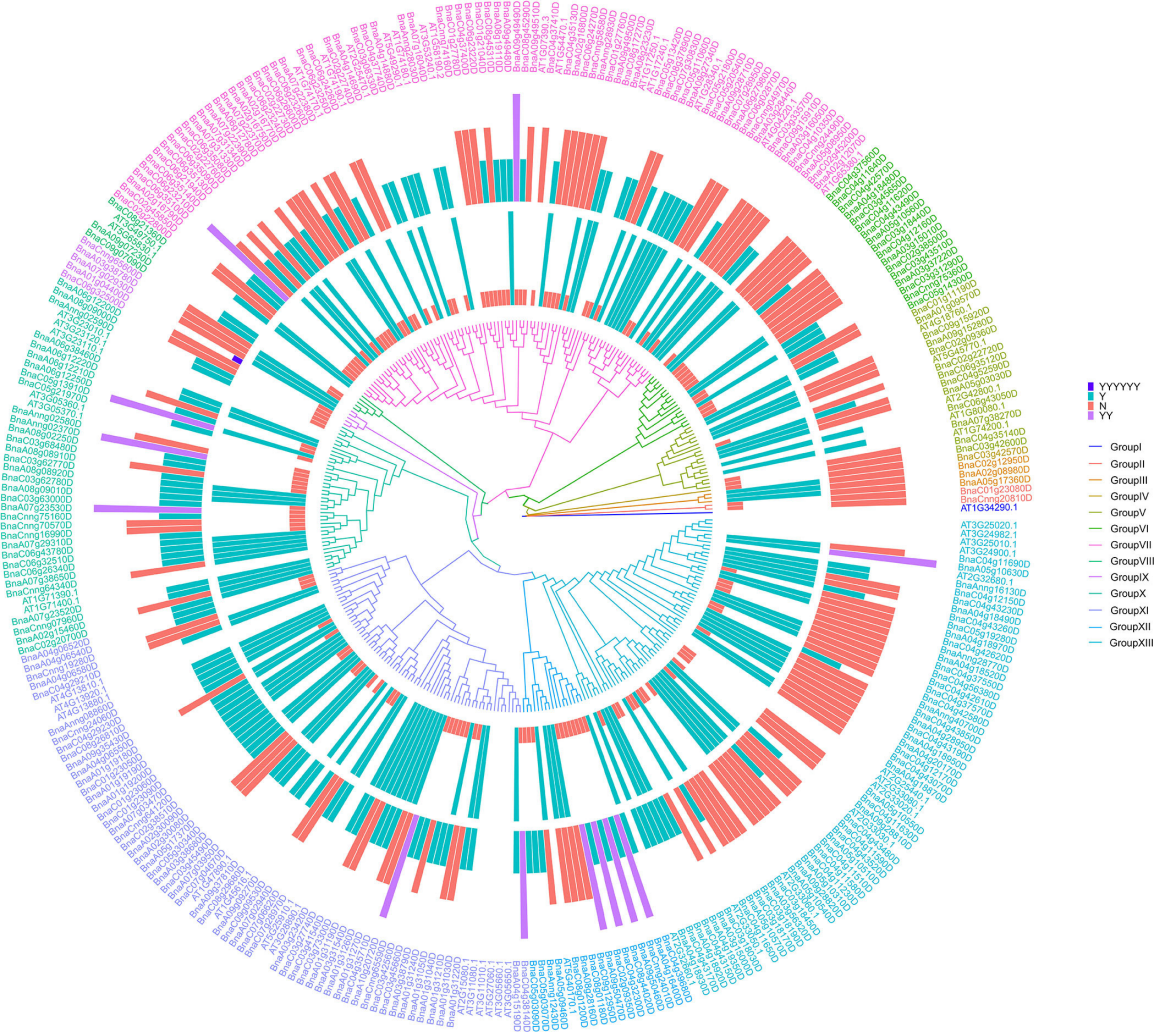
Phylogenetic trees of RLP proteins in *Arabidopsis thaliana* and *Brassica napus*. The phylogenetic tree was generated by the ML method with bootstrap analysis (1,000 bootstrap replicates) using an amino acid sequence alignment of RLP proteins from *A. thaliana* and *B. napus* by the MEGA 7.0 program. RLP proteins were clustered into 13 subgroups. The rings surrounded by the phylogenetic tree stand for two types of domains. The inner one represents the transmembrane domain and the outer represents the signal peptide. “Y” means that RLP contains one transmembrane domain/signal peptide, “YY” for two transmembrane domains, and “YYYYYY” for six transmembrane domains. “N” means that RLP does not contain transmembrane domain/signal peptide.

To better understand the evolving relationship between *Bn*RLPs and *At*RLPs, a phylogenetic tree was constructed by the ML method (Figure 4). The RLP proteins were clustered into 13 subgroups. Group I to Group IV consisted of no more than three proteins, respectively. The protein constructs of most *Bn*RLPs in GroupV, X, and XI were canonical. More than half of *Bn*RLPs in GroupVI had an SP domain or a TM domain. Most *Bn*RLPs possessed only one type of domain, and two TM domains were predicted in four *Bn*RLPs in GroupXII. In addition, besides a sub-clade of *Bn*RLPs that only possessed the TM domain (one or more), the quantity of *Bn*RLPs that the missing TM domain had increased to 69.23% in GroupXII, but most had an SP domain. Some *RLP* genes in GroupVII were more conserved since most members had no introns. Some bunches of tandem *AtRLPs* were especially prone to cluster together, which evolved independently with *BnRLPs* in Group XI and Group XIII. Other tandem ones evolved together with *BnRLPs*, like *At1g17240* (*AtRLP2*) and *At1g17250* (*AtRLP3*) in GroupVII, *At2g32660* (*AtRLP22*) and *At2g32680* (*AtRLP23*) in GroupXIII.

### Analysis of *cis*-acting elements in the promoter region of *BnRLPs*

**Figure 5:**
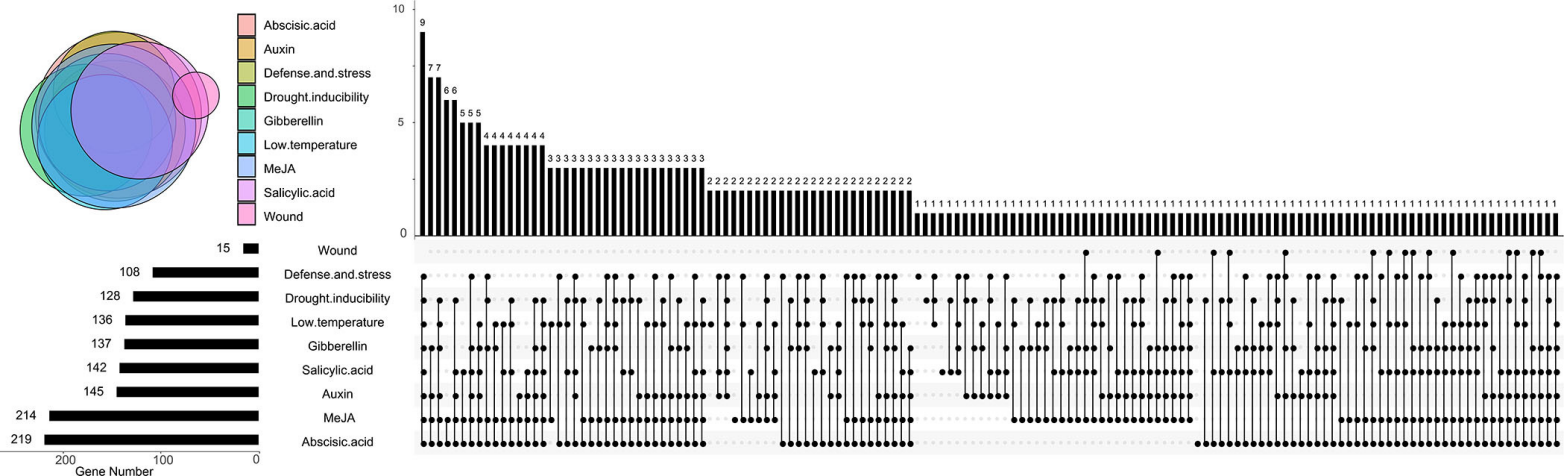
Distributions of *cis*-acting elements in *BnRLP* promoter. The Upset plot shows the distribution of *cis*-acting elements in *BnRLP* promoter. The bar chart above represents the number of genes contained in each type of RLP. The bar chart at the bottom left represents the number of genes included in each type of *cis*-acting element. The dotted line shows the types of *cis*-acting elements contained in the group.

*Cis*-acting elements that are distributed in the promoter region can help predict the function of candidate genes. Most *At*RLPs and *Bn*RLPs were identified as related to stress responses, so the *cis*-acting elements related to stresses were further analyzed (Supplementary Figure 5, Figure 5). Concretely, *cis*-acting elements were categorized into MeJA-responsive (MeJA, name after), gibberellin-responsive (GA), abscisic acid-responsive (ABA), drought-inducible (drought), auxin-responsive (auxin), low temperature-responsive (low temperature), salicylic acid-responsive (SA), wound-responsive (wound), and defense- and stress-responsive (defense and stress) related. Approximately, 79.3 and 77.5% of the promoters contained MeJA and ABA-related *cis*-acting elements, followed by GA- and wound-related, which were found in half of the promoters of *BnRLPs*. Only 15 *BnRLPs* were related to wound responsiveness. Additionally, a quarter contained no more than three types of *cis*-acting elements. Among them, seven *BnRLPs* might exhibit functional specificity since they possessed single type of *cis*-acting element, such as *BnaA09g37810D* (drought), *BnaA02g12070D* (SA), *BnaC02g20700D* and *BnaC09g12950D* (MeJA), *BnaC02g45850D* and *BnaC04g12150D* (low temperature), *BnaC09g15910D* (defense and stress), and *BnaC04g52590D* (ABA).

### Expression pattern of *RLP* genes induced *by Sclerotinia sclerotiorum*

**Figure 6:**
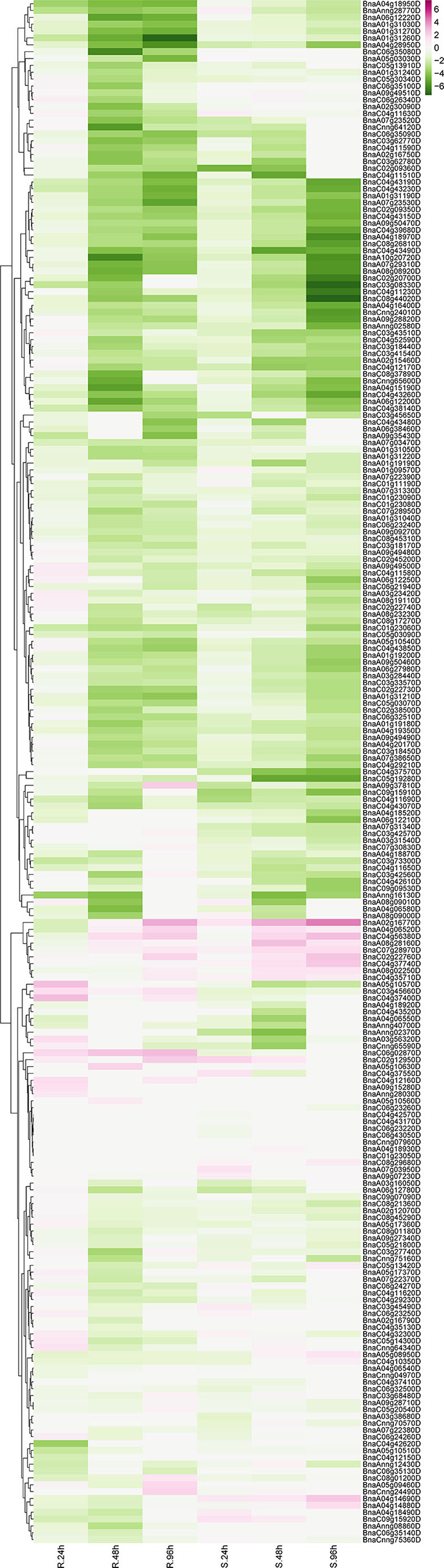
The expression heat map of *BnRLP* genes elicited by *S. sclerotiorum*. The expression heat map of *BnRLP* genes in the resistant (R) and susceptible (S) *Brassica napus* alleles at 24, 48, and 96 h post-inoculation, TPM value derived from the GEO database was used for heat map construction.

*Sclerotinia sclerotiorum* is one of the most common pathogens worldwide, which is the causal agent of stem rot disease in *Brassica* crops causing devastating decline in the economic value. To further unveil the protagonists in pathogen-defense responses, expression profiling data of *RLP* genes were derived from the GEO database. One series (accession No. SRP053361) (Wu et al., 2016b) was downloaded and analyzed. In total, 225 *BnRLP* genes identified showed expressional fluctuations during 24, 48, and 96 hpi (hour post-inoculated) by *S. sclerotiorum* in resistance (R) or susceptible (S) rapeseeds (Figure 6). To confirm the reliability of the dataset and further identify the genes that can be induced by *S. sclerotiorum*, seven upregulated *BnRLP* gene expressions were detected using real-time fluorescent quantitative PCR (Supplementary Figure 6). As we observed, besides inflorescence, the plant stem and leaves were also susceptible to *S. sclerotiorum*, and the infected stem becomes more prone to lodging at the flowering stage, so the fact that the RLPs are induced at a higher expression level at the flowering stage might have the potential to possess greater agricultural applicability. To this end, the stem and leaves were selected as target tissues to illuminate the tissue specificity under *S. sclerotiorum* infection. The results showed that those *RLP* genes can be induced significantly by *S. sclerotiorum*, but varied in expression level and pattern. Among them, *BnaC07g28970D, BnaC02g22760D*, and *BnaA02g16770D* accumulated more transcripts in the stem than that in the leaf, and the differences ranged from 2- to 4-fold. On the contrary, *BnaA08g02250D, BnaA08g28160D, BnaC04g56380D*, and *BnaA04g14880D* were induced more in the leaf. Moreover, *BnaA03g56320D* showed a similar expression level in stem and leaf, but at a relatively lower level compared to other genes. Overall, the results showed that all selected upregulated genes derived from the database could be induced by *S. sclerotiorum*.

**Figure 7:**
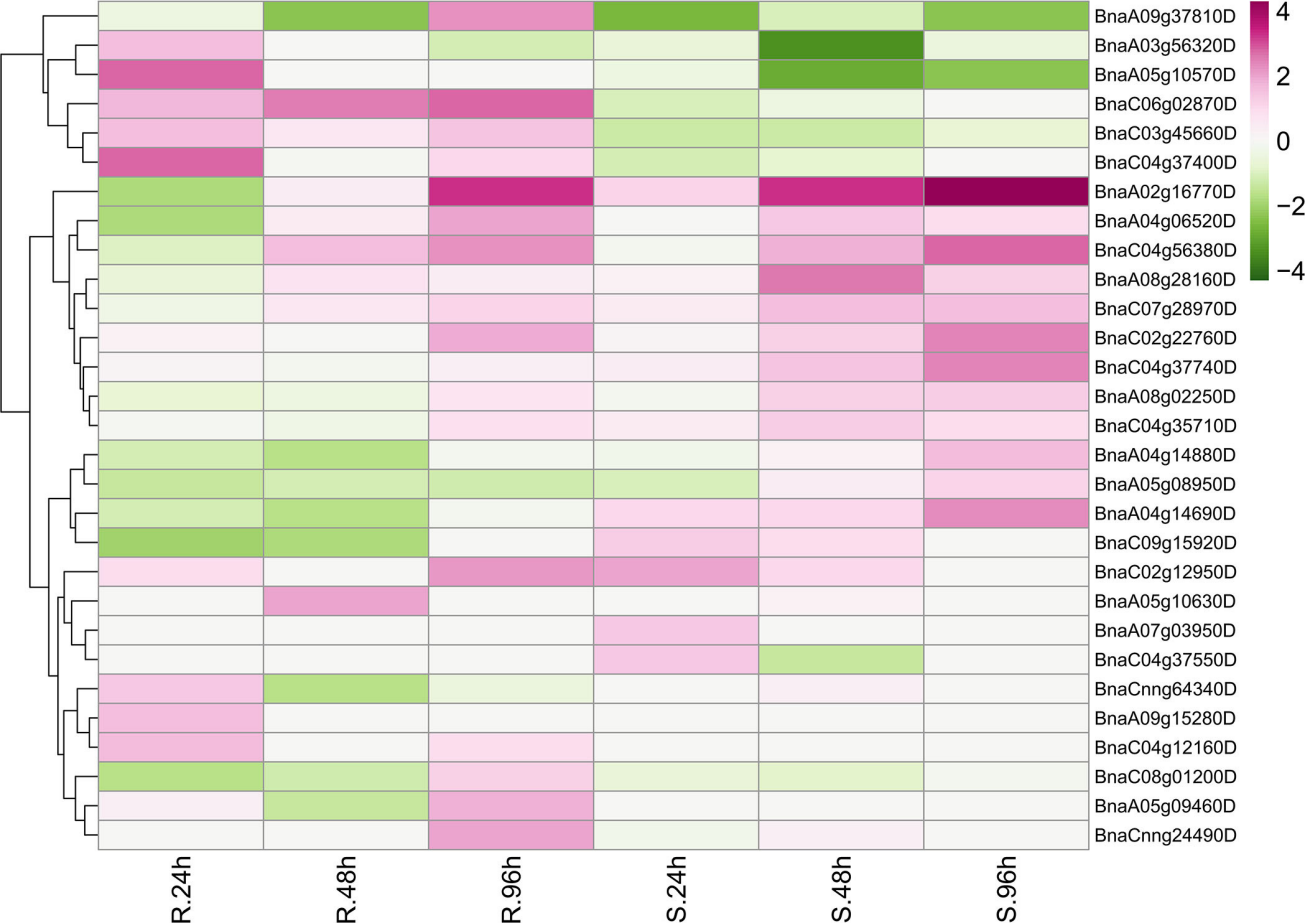
Upregulated *BnRLP*s elicited by *Sclerotinia sclerotiorum*. The expression heat map of upregulated *BnRLP* genes in the resistant (R) and susceptible (S) *B. napus* alleles at 24, 48, and 96 h post-inoculation by *S. sclerotiorum*, TPM value derived from the GEO database was used for heat map construction.

Unexpectedly, only 29 *BnRLPs* were upregulated (more than two times compared to mock) at a certain time point (Figure 7). Among them, some *BnRLPs* presented expression specificity between R and S, such as four *BnRLPs* (*BnaA04g14880D, BnaA05g08950D, BnaA04g14690D, BnaC09g15920D*) that were induced merely in S, and six *BnRLPs* (*BnaA09g37810D, BnaA03g56320D, BnaA05g10570D, BnaC06g02870D, BnaC03g45660D*, and *BnaC04g37400D*) in R. The expressional differences of some *BnRLPs* between R and S appeared due to the inconsistency of the induction time. For instance, transcript accumulations of *BnaC07g28970D, BnaC02g22760D*, and *BnaA02g16770D* in R were slower than S, and their expression levels at 96 hpi (R) were approximately equal to that at 48 hpi (S). Additionally, as results show, *BnaC02g22760D* and *BnaA02g16770D* were induced from 24hpi in S, and their expression levels continued increasing even at 96 hpi reaching a level of 13.85 and 31.03 times, respectively, but they were induced from 96 hpi in R. *BnaA08g28160D* and *BnaC04g56380D* possessed similar expression patterns between R and S. The transcript accumulations of *BnaA08g28160D* reached a peak at 48 hpi in both R and S. Furthermore, *BnaC04g56380D* was induced at 48 hpi and kept accumulating until 96 hpi in two rapeseed ecotypes. It is worth mentioning that *LepR3*/*Rlm2* encodes a receptor-like protein triggered by the *L. maculans* effector AVRLM1 (Larkan et al., 2013, 2015), but the expression of *BnaA10g20720D*, which possessed 100% identity and was co-located in the same genetic interval with *LepR3*/*Rlm2*, was inhibited by *S. sclerotiorum* both in R and S (Figure 6).

## Discussion

The fluctuation of gene expression profiles induced by various pathogens can be used as an indicator for functional assumption and further characterization. To investigate the possible functions of *BnRLP*s, we compared the transcriptional profiles of *RLP* genes with that of the functional assigned *AtRLP* genes. Intriguingly, *AtRLP10* (*CLV2, AT1G65380.1*) was identified as a regulator of plant meristem and organ development (Jeong et al., 1999; Wang et al., 2011). As its homologs, the transcript abundance of *BnaC02g45200D* and *BnaA02g12070D* were decreased when there was a stimulus exerted by *S. sclerotiorum*. The expressional inclination of *RLP10* genes indicated that their functions might be conserved among *Brassica* species and *A. thaliana* due to the trade-off between growth and defense response, which is widely known in plants. Another hint in favor of the possibility of functional conservation among *RLP10* genes is that they shared high similarity/identity with *AtRLP10* no matter in sequence (CDS and protein) or structural layer (Supplementary Figure 7). Unlike *RLP10*, a functional divergence occurred among the *RLP30* genes as *AtRLP30* (*AT3G05360.1*) was suggested to be involved in resistance toward *S. sclerotiorum*, while *BnRLP30* genes (*BnaAnng02580D* and *BnaAnng02370D*) were not induced by it (Figure 6). The low similarity/identity between *BnRLP30s* and *AtRLP30* also supported the probability of functional divergence in *B. napus* (Supplementary Figure 7). Instead, *BnaA02g16770D* and *BnaC02g22760D*, homologs of *AtRLP15* (*AT1G74190.1*), might be fabulous candidates for *S. sclerotiorum* resistance research since they were proven to be few but induced to a greater extent.

The case that one single *RLP* gene is transcriptionally regulated by multiple external stimuli and involved in multiple biological processes has been reported in *A. thaliana* (Wu et al., 2016a). It was also a common phenomenon in *B. napus*, as exemplified by *BnaA09g09270D*, which can be induced by both *S. sclerotiorum* and *L. maculans* (Becker et al., 2017; Duke et al., 2017). The comparative analysis of *BnRLP*s phylogeny and their transcriptional profiles indicated that there was no significant correlation between *BnRLP*s phylogeny (predicted by sequence similarity) and the expression patterns, and this is in line with previous publications (Steidele and Stam, 2021). On the contrary, the diversification of gene expression patterns was observed among the duplicated *BnRLP*s paralogs. Gene duplication widely exists during the process of plant evolution in multiple forms, like tandem duplication, segmental duplication, and whole-genome duplication (Kondrashov et al., 2002; Conant and Wolfe, 2008). Retained duplicated genes can provide raw genetic resources for functional innovation, and the novel function can derive from several different ways, including gain-of-function mutations, the subdivision of ancestral functions, and gene dosage changes (Conant and Wolfe, 2008). In this study, tandem duplication was commonly found in *BnRLP*s, and some *BnRLP*s were amplified by a wide margin when compared with their counterparts in *A. thaliana*, such as homologs of *AtRLP27* (*AT2G33060.1*) and *AtRLP54* (*AT5G40170.1*).

As *B. napus* was hybridized by *B. rapa* and *B. oleracea*, in general, the evolution of BnRLP proteins was relatively conserved among the three *Brassica* species. However, because of gene duplications and translocations after hybridization, inaccurate assembly universally occurred during genetic recombination in plants, resulting in the loss-of-function of some genes (Schnable et al., 2012; Boutte et al., 2020). In this study, 5, 10, and 24 proteins were detected as containing no LRR domains in *B. rapa, B. oleracea*, and *B. napus*, respectively, but remained as RLPs. They possessed a high sequence identity and had a similar protein construct (consisted of SP and/or TM domain) with other typical RLPs. So those proteins with no LRR domains were also included to better reveal the evolution of BnRLPs.

When we did the phylogenetic analysis, the phylogeny of *At*RLPs resembles that performed by previous publications (Wang et al., 2008; Steidele and Stam, 2021). In line with the classification by Steidele and Stam (2021), most upregulated *Bn*RLPs were homologous with *At*RLPs that were clustered into pathogen-responsive RLP clade. The exception is that *BnaA02g16770D* and *BnaC02g22760D*, homologs of *AtRLP15* (*AT1G74190.1*) which were grouped into a basal sub-family but not the aforementioned pathogen-responsive RLP clade, upregulated after pathogen treatment to a great extent.

Based on the dataset, six *Bn*RLP genes, namely *BnaA09g37810D, BnaA03g56320D, BnaA05g10570D, BnaC06g02870D, BnaC03g45660D*, and *BnaC04g37400D*, upregulated specifically in resistant rapeseed by *S. sclerotiorum* elicitation. Correspondingly, four *Bn*RLP*s* (*BnaA04g14880D, BnaA05g08950D, BnaA04g14690D*, and *BnaC09g15920D*) showed preference for susceptible rapeseed. Those genes identified from incompatible interactions might be good candidates for further functional assignment of RLP genes against *S. sclerotiorum* infection. *BnaA02g16770D* and *BnaC02g22760D*, which could be induced universally by *S. sclerotiorum*, might also play a role in preventing fungal infection. Zhongyou821 was identified as a mid-resistant *B.napus* variety (Wang et al., 2004) and commonly used as a control cultivar for investigating disease indexes. In this study, we also used Zhongyou821 for infection and performed qRT-PCR to verify the expression level of some upregulated *BnRLP* genes, and the result showed that the transcript abundance in this study was dozens of times than that derived from the RNAseq datasets, but further confirmed they could be induced by *S. sclerotiorum*. The large difference might be due to the selection of different cultivars or ascribed to spatiotemporal expression differences. Overall, all selected upregulated *BnRLPs* genes derived from the database could be induced by *S. sclerotiorum* in Zhongyou821.

## Conclusion

We conducted identification, gene and protein characterization, localization, evolution, and expression analysis of the highly conserved RLP members in *B. napus* and found that a majority of RLP gene families in this study were relatively conserved during the evolution of *Brassicaceae*. Exploring effective resistance genes against a vast variety of microbial pathogens is critical for crop breeding. Genome-wide identification and expression analysis of the RLP family members in *B. napus* provided an alternative strategy to reinforce the resistance against major pathogens in *Brassicaceae*. Our results provide important clues for further investigations of the function of *Brassicaceae* RLPs involved in the development and immune response and pave the way to molecular breeding of disease-resistant rapeseed.

## Data availability statement

The datasets presented in this study can be found in online repositories. The names of the repository/repositories and accession number(s) can be found in the article/Supplementary material.

## Author contributions

WL: formal analysis, writing the original draft, data curation, and validation. JL: investigation, resources, and visualization. CY: data curation. SX: conceptualization, writing—review and editing, and supervision. All authors contributed to the article and approved the submitted version.

## Funding

This work was supported by grants from the National Natural Science Foundation of China (Grants 31971836 and 30970247), the Hunan Province Natural Science Fund for Distinguished Young Scholars (Grant 11JJ1007), the Chongqing municipal education commission (Grant KJQN201900533), and the Chongqing Bureau of Human Resources and Social Security Post-doctoral Funding (0019).

## Conflict of interest

The authors declare that the research was conducted in the absence of any commercial or financial relationships that could be construed as a potential conflict of interest.

## Publisher's note

All claims expressed in this article are solely those of the authors and do not necessarily represent those of their affiliated organizations, or those of the publisher, the editors and the reviewers. Any product that may be evaluated in this article, or claim that may be made by its manufacturer, is not guaranteed or endorsed by the publisher.

## Supplementary material

Supplementary Table 1 *RLP* gene information in *B. napus, B. rapa* and *B. oleracea*.

Supplementary Table 2 Ka/Ks in *A.thaliana* and *B. napus, B. rapa* and *B. oleracea* pairs.

Supplementary Table 3 Primers in this study.

Supplementary Figure 1 Gene and protein structures of *RLP* genes in *B. napus*. The green bar represents CDS, the black line represents intron, and the blue bar represents UTR. The pink bar stands for the LRR motif, the yellow bar for the transmembrane domain, and the red for the signal peptide.

Supplementary Figure 2 Motif and conserved domain of RLP in *B. napus*. Motif labeled as motif 1-motif 10. The green bar represents CDS, the blue bar represents UTR. The yellow bar stands for the conserved domain PLN03150 and the pink bar for PLN00113.

Supplementary Figure 3 Motifs in *Bn*RLPs.

Supplementary Figure 4 Syntenic relationship of RLP-encoding genes between *A. thaliana* and *Brassicaceae* species.

Supplementary Figure 5 *Cis*-acting element in *BnRLP* promoter.

Supplementary Figure 6 Tissue specificity of upregulated *BnRLP*s under *S. sclerotiorum* infection. The box plot represents dispersion and expression degree. Gene expression was detected with at least three biological repeats and three experiment repeats, and statistical analysis showed that compared with controls, the expression of all genes reached significant levels when elicited by *S. sclerotiorum* (*p*-value < 0.05).

Supplementary Figure 7 Protein similarity and identity of RLP families. Data on the upper right present the protein sequence identity, and data on the bottom left present the protein sequence similarity.

## References

[B1] Albert I. Böhm H. Albert M. Feiler C. E. Imkampe J. Wallmeroth N.. (2015). An RLP23-SOBIR1-BAK1 complex mediates NLP-triggered immunity. Nat Plants 1, 15140. 10.1038/nplants.2015.140 27251392

[B2] Bailey T. L. Elkan C. (1994). Fitting a mixture model by expectation maximization to discover motifs in biopolymers. Proc. Int. Conf. Intell. Syst. Mol. Biol. 2, 28–36. 7584402

[B3] Bayer P. E. Golicz A. A. Tirnaz S. Chan C.-K. K. Edwards D. Batley J. (2019). Variation in abundance of predicted resistance genes in the *Brassica oleracea* pangenome. Plant Biotechnol. J. 17, 789–800. 10.1111/pbi.13015 30230187 PMC6419861

[B4] Becker M. G. Zhang X. Walker P. L. Wan J. C. Millar J. L. Khan D.. (2017). Transcriptome analysis of the *Brassica napus*-*Leptosphaeria maculans* pathosystem identifies receptor, signaling and structural genes underlying plant resistance. Plant J. 90, 573–586. 10.1111/tpj.13514 28222234

[B5] Belfanti E. Silfverberg-Dilworth E. Tartarini S. Patocchi A. Barbieri M. Zhu J.. (2004). The HcrVf2 gene from a wild apple confers scab resistance to a transgenic cultivated variety. Proc. Natl. Acad. Sci. USA. 101, 886–890. 10.1073/pnas.0304808101 14715897 PMC321776

[B6] Boutte J. Maillet L. Chaussepied T. Letort S. Aury J.-M. Belser C.. (2020). Genome size variation and comparative genomics reveal intraspecific diversity in *Brassica rapa*. Front. Plant Sci. 11, 577536. 10.3389/fpls.2020.577536 33281844 PMC7689015

[B7] Bustin S. A. Benes V. Garson J. A. Hellemans J. Huggett J. Kubista M.. (2009). The MIQE guidelines: minimum information for publication of quantitative real-time PCR experiments. Clin. Chem. 55, 611–622. 10.1373/clinchem.2008.112797 19246619

[B8] Catanzariti A.-M. Do H. T. T. Bru P. de Sain M. Thatcher L. F. Rep M.. (2017). The tomato I gene for *Fusarium wilt* resistance encodes an atypical leucine-rich repeat receptor-like protein whose function is nevertheless dependent on SOBIR1 and SERK3/BAK1. Plant J. 89, 1195–1209. 10.1111/tpj.13458 27995670

[B9] Chen C. Chen H. Zhang Y. Thomas H. R. Frank M. H. He Y.. (2020). TBtools: an integrative toolkit developed for interactive analyses of big biological data. Mol. Plant 13, 1194–1202. 10.1016/j.molp.2020.06.009 32585190

[B10] Chen H. Wang T. He X. Cai X. Lin R. Liang J.. (2022). BRAD V3.0: an upgraded Brassicaceae database. Nucleic Acids Res. 50, D1432–D1441. 10.1093/nar/gkab1057 34755871 PMC8728314

[B11] Chen I.-H. Huang Y.-P. Tseng C.-H. Ni J.-T. Tsai C.-H. Hsu Y.-H.. (2017). *Nicotiana benthamiana* elicitor-inducible leucine-rich repeat receptor-like protein assists bamboo mosaic virus cell-to-cell movement. Front. Plant Sci. 8, 1736. 10.3389/fpls.2017.01736 29056941 PMC5635722

[B12] Chen J.-Y. Huang J.-Q. Li N.-Y. Ma X.-F. Wang J.-L. Liu C.. (2015). Genome-wide analysis of the gene families of resistance gene analogues in cotton and their response to *Verticillium wilt*. BMC Plant Biol. 15, 148. 10.1186/s12870-015-0508-3 26084488 PMC4471920

[B13] Chen X. Truksa M. Shah S. Weselake R. J. (2010). A survey of quantitative real-time polymerase chain reaction internal reference genes for expression studies in *Brassica napus*. Anal. Biochem. 405, 138–140. 10.1016/j.ab.2010.05.032 20522329

[B14] Conant G. C. Wolfe K. H. (2008). Turning a hobby into a job: how duplicated genes find new functions. Nat. Rev. Genet. 9, 938–950. 10.1038/nrg2482 19015656

[B15] Deng W. Wang Y. Liu Z. Cheng H. Xue Y. (2014). HemI: a toolkit for illustrating heatmaps. PLoS ONE 9, e111988. 10.1371/journal.pone.0111988 25372567 PMC4221433

[B16] Duke K. A. Becker M. G. Girard I. J. Millar J. L. Dilantha Fernando W. G. Belmonte M. F.. (2017). The biocontrol agent *Pseudomonas chlororaphis* PA23 primes *Brassica napus* defenses through distinct gene networks. BMC Genomics 18, 467. 10.1186/s12864-017-3848-6 28629321 PMC5477169

[B17] Fan L. Fröhlich K. Melzer E. Pruitt R. N. Albert I. Zhang L.. (2022). Genotyping-by-sequencing-based identification of *Arabidopsis* pattern recognition receptor RLP32 recognizing proteobacterial translation initiation factor IF1. Nat. Commun. 13, 1294. 10.1038/s41467-022-28887-4 35277499 PMC8917236

[B18] Fritz-Laylin L. K. Krishnamurthy N. Tör M. Sjölander K. V. Jones J. D. G. (2005). Phylogenomic analysis of the receptor-like proteins of rice and *Arabidopsis*. Plant Physiol. 138, 611–623. 10.1104/pp.104.054452 15955925 PMC1150382

[B19] Hu B. Jin J. Guo A. Zhang H. Luo J. Gao G. (2015). GSDS 2.0: an upgraded gene feature visualization server. Bioinformatics 31, 1296–1297. 10.1093/bioinformatics/btu817 25504850 PMC4393523

[B20] Jehle A. K. Fürst U. Lipschis M. Albert M. Felix G. (2013a). Perception of the novel MAMP eMax from different *Xanthomonas* species requires the *Arabidopsis* receptor-like protein ReMAX and the receptor kinase SOBIR. Plant Signal. Behav. 8, e27408. 10.4161/psb.27408 24384530 PMC4091347

[B21] Jehle A. K. Lipschis M. Albert M. Fallahzadeh-Mamaghani V. Fürst U. Mueller K.. (2013b). The receptor-like protein ReMAX of *Arabidopsis* detects the microbe-associated molecular pattern eMax from *Xanthomonas*. Plant Cell 25, 2330–2340. 10.1105/tpc.113.110833 23898033 PMC3723629

[B22] Jeong S. Trotochaud A. E. Clark S. E. (1999). The *Arabidopsis* CLAVATA2 gene encodes a receptor-like protein required for the stability of the CLAVATA1 receptor-like kinase. Plant Cell 11, 1925–1934. 10.1105/tpc.11.10.1925 10521522 PMC144110

[B23] Jones D. A. Thomas C. M. Hammond-Kosack K. E. Balint-Kurti P. J. Jones J. D. (1994). Isolation of the tomato Cf-9 gene for resistance to *Cladosporium fulvum* by transposon tagging. Science 266, 789–793. 10.1126/science.7973631 7973631

[B24] Kang W.-H. Yeom S.-I. (2018). Genome-wide identification, classification, and expression analysis of the receptor-like protein family in tomato. Plant Pathol. J. 34, 435–444. 10.5423/PPJ.OA.02.2018.0032 30369853 PMC6200040

[B25] Kawchuk L. M. Hachey J. Lynch D. R. Kulcsar F. van Rooijen G. Waterer D. R.. (2001). Tomato Ve disease resistance genes encode cell surface-like receptors. Proc. Natl. Acad. Sci. USA. 98, 6511–6515. 10.1073/pnas.091114198 11331751 PMC33499

[B26] Kondrashov F. A. Rogozin I. B. Wolf Y. I. Koonin E. V. (2002). Selection in the evolution of gene duplications. Genome Biol. 3, RESEARCH0008. 10.1186/gb-2002-3-2-research0008 11864370 PMC65685

[B27] Kourelis J. van der Hoorn R. A. L. (2018). Defended to the nines: 25 years of resistance gene cloning identifies nine mechanisms for R protein function. Plant Cell 30, 285–299. 10.1105/tpc.17.00579 29382771 PMC5868693

[B28] Larkan N. J. Lydiate D. J. Parkin I. A. P. Nelson M. N. Epp D. J.. (2013). The *Brassica napus* blackleg resistance gene LepR3 encodes a receptor-like protein triggered by the *Leptosphaeria maculans* effector AVRLM1. New Phytol. 197, 595–605. 10.1111/nph.12043 23206118

[B29] Larkan N. J. Ma L. Borhan M. H. (2015). The *Brassica napus* receptor-like protein RLM2 is encoded by a second allele of the LepR3/Rlm2 blackleg resistance locus. Plant Biotechnol. J. 13, 983–992. 10.1111/pbi.12341 25644479

[B30] Lescot M. Déhais P. Thijs G. Marchal K. Moreau Y. Van de Peer Y.. (2002). PlantCARE, a database of plant cis-acting regulatory elements and a portal to tools for *in silico* analysis of promoter sequences. Nucleic Acids Res. 30, 325–327. 10.1093/nar/30.1.325 11752327 PMC99092

[B31] Li W. Lu J. Lu K. Yuan J. Huang J. Du H.. (2016). Cloning and Phylogenetic analysis of *Brassica napus* L. caffeic acid o-methyltransferase 1 gene family and its expression pattern under drought stress. PLoS ONE 11, e0165975. 10.1371/journal.pone.0165975 27832102 PMC5104432

[B32] Liebrand T. W. H. van den Berg G. C. M. Zhang Z. Smit P. Cordewener J. H. G. America A. H. P.. (2013). Receptor-like kinase SOBIR1/EVR interacts with receptor-like proteins in plant immunity against fungal infection. Proc. Natl. Acad. Sci. USA. 110, 10010–10015. 10.1073/pnas.1220015110 23716655 PMC3683720

[B33] Ma L. Borhan M. H. (2015). The receptor-like kinase SOBIR1 interacts with *Brassica napus* LepR3 and is required for *Leptosphaeria maculans* AvrLm1-triggered immunity. Front. Plant Sci. 6, 933. 10.3389/fpls.2015.00933 26579176 PMC4625043

[B34] Malnoy M. Xu M. Borejsza-Wysocka E. Korban S. S. Aldwinckle H. S. (2008). Two receptor-like genes, Vfa1 and Vfa2, confer resistance to the fungal pathogen *Venturia inaequalis* inciting apple scab disease. Mol. Plant Microbe Interact. 21, 448–458. 10.1094/MPMI-21-4-0448 18321190

[B35] Nadeau J. A. Sack F. D. (2002). Control of stomatal distribution on the *Arabidopsis* leaf surface. Science 296, 1697–1700. 10.1126/science.1069596 12040198

[B36] Nagaharu U. (1935). Genome analysis in *Brassica* with special reference to the experimental formation of *B. napus* and peculiar mode of fertilization. Japanese Journal of Botany 7, 389–452.

[B37] Ono E. Mise K. Takano Y. (2020). RLP23 is required for *Arabidopsis* immunity against the grey mould pathogen *Botrytis cinerea*. Sci. Rep. 10, 13798. 10.1038/s41598-020-70485-1 32796867 PMC7428006

[B38] Petre B. Hacquard S. Duplessis S. Rouhier N. (2014). Genome analysis of poplar LRR-RLP gene clusters reveals RISP, a defense-related gene coding a candidate endogenous peptide elicitor. Front. Plant Sci. 5, 111. 10.3389/fpls.2014.00111 24734035 PMC3975113

[B39] Ramonell K. Berrocal-Lobo M. Koh S. Wan J. Edwards H. Stacey G.. (2005). Loss-of-function mutations in chitin responsive genes show increased susceptibility to the powdery mildew pathogen *Erysiphe cichoracearum*. Plant Physiol. 138, 1027–1036. 10.1104/pp.105.060947 15923325 PMC1150417

[B40] Ron M. Avni A. (2004). The receptor for the fungal elicitor ethylene-inducing xylanase is a member of a resistance-like gene family in tomato. Plant Cell 16, 1604–1615. 10.1105/tpc.022475 15155877 PMC490049

[B41] Schnable J. C. Freeling M. Lyons E. (2012). Genome-wide analysis of syntenic gene deletion in the grasses. Genome Biol. Evol. 4, 265–277. 10.1093/gbe/evs009 22275519 PMC3318446

[B42] Shen Y. Diener A. C. (2013). *Arabidopsis thaliana* resistance to fusarium oxysporum 2 implicates tyrosine-sulfated peptide signaling in susceptibility and resistance to root infection. PLoS Genet. 9, e1003525. 10.1371/journal.pgen.1003525 23717215 PMC3662643

[B43] Steidele C. E. Stam R. (2021). Multi-omics approach highlights differences between RLP classes in *Arabidopsis thaliana*. BMC Genomics 22, 557. 10.1186/s12864-021-07855-0 34284718 PMC8290556

[B44] Taguchi-Shiobara F. Yuan Z. Hake S. Jackson D. (2001). The fasciated ear2 gene encodes a leucine-rich repeat receptor-like protein that regulates shoot meristem proliferation in maize. Genes Dev. 15, 2755–2766. 10.1101/gad.208501 11641280 PMC312812

[B45] Tirnaz S. Bayer P. E. Inturrisi F. Zhang F. Yang H. Dolatabadian A.. (2020). Resistance gene analogs in the brassicaceae: identification, characterization, distribution, and evolution. Plant Physiol. 184, 909–922. 10.1104/pp.20.00835 32796089 PMC7536671

[B46] Wang G. Ellendorff U. Kemp B. Mansfield J. W. Forsyth A. Mitchell K.. (2008). A genome-wide functional investigation into the roles of receptor-like proteins in *Arabidopsis*. Plant Physiol. 147, 503–517. 10.1104/pp.108.119487 18434605 PMC2409048

[B47] Wang G. Long Y. Thomma B. P. H. J. de Wit P. J. G. M. Angenent G. C. Fiers M. (2010). Functional analyses of the CLAVATA2-like proteins and their domains that contribute to CLAVATA2 specificity. Plant Physiol. 152, 320–331. 10.1104/pp.109.148197 19897604 PMC2799366

[B48] Wang G. Zhang Z. Angenent G. C. Fiers M. (2011). New aspects of CLAVATA2, a versatile gene in the regulation of *Arabidopsis* development. J. Plant Physiol. 168, 403–407. 10.1016/j.jplph.2010.08.015 20961653

[B49] Wang H. Z. Liu G. H. Zheng Y. B. Wang X. F. Yang Q. (2004). Breeding of the *Brassica napus* cultivar Zhongshuang 9 with high-resistance to *Sclerotinia sclerotiorum* and dynamics of its important defense enzyme activity. Sci. Agric. Sin. 37, 23–28.

[B50] Wang Y. Tang H. Debarry J. D. Tan X. Li J. Wang X.. (2012). MCScanX: a toolkit for detection and evolutionary analysis of gene synteny and collinearity. Nucleic Acids Res. 40, e49. 10.1093/nar/gkr1293 22217600 PMC3326336

[B51] Wolf S. van der Does D. Ladwig F. Sticht C. Kolbeck A. Schürholz A.-K.. (2014). A receptor-like protein mediates the response to pectin modification by activating brassinosteroid signaling. Proc. Natl. Acad. Sci. USA. 111, 15261–15266. 10.1073/pnas.1322979111 25288746 PMC4210321

[B52] Wu J. Liu Z. Zhang Z. Lv Y. Yang N. Zhang G.. (2016a). Transcriptional regulation of receptor-like protein genes by environmental stresses and hormones and their overexpression activities in *Arabidopsis thaliana*. J. Exp. Bot. 67, 3339–3351. 10.1093/jxb/erw152 27099374 PMC4892725

[B53] Wu J. Zhao Q. Yang Q. Liu H. Li Q. Yi X.. (2016b). Comparative transcriptomic analysis uncovers the complex genetic network for resistance to *Sclerotinia sclerotiorum* in *Brassica napus*. Sci. Rep. 6, 19007. 10.1038/srep19007 26743436 PMC4705546

[B54] Yang H. Bayer P. E. Tirnaz S. Edwards D. Batley J. (2020). Genome-wide identification and evolution of receptor-like kinases (RLKs) and receptor like proteins (RLPs) in *Brassica juncea*. Biology 10, 17. 10.3390/biology10010017 33396674 PMC7823396

[B55] Yang Y. Chen T. Ling X. Ma Z. (2017). Gbvdr6, a gene encoding a receptor-like protein of cotton (*Gossypium barbadense*), confers resistance to verticillium wilt in *Arabidopsis* and upland cotton. Front. Plant Sci. 8, 2272. 10.3389/fpls.2017.02272 29387078 PMC5776133

[B56] Zhang B. Li P. Su T. Li P. Xin X. Wang W.. (2018). BrRLP48, encoding a receptor-like protein, involved in downy mildew resistance in *Brassica rapa*. Front. Plant Sci. 9, 1708. 10.3389/fpls.2018.01708 30532761 PMC6265505

[B57] Zhang L. Kars I. Essenstam B. Liebrand T. W. H. Wagemakers L. Elberse J.. (2014). Fungal endopolygalacturonases are recognized as microbe-associated molecular patterns by the *Arabidopsis* receptor-like protein RESPONSIVENESS TO BOTRYTIS POLYGALACTURONASES1. Plant Physiol. 164, 352–364. 10.1104/pp.113.230698 24259685 PMC3875813

[B58] Zhang W. Fraiture M. Kolb D. Löffelhardt B. Desaki Y. Boutrot F. F. G.. (2013). *Arabidopsis* receptor-like protein30 and receptor-like kinase suppressor of BIR1-1/EVERSHED mediate innate immunity to necrotrophic fungi. Plant Cell 25, 4227–4241. 10.1105/tpc.113.117010 24104566 PMC3877809

[B59] Zhang Y. Yang Y. Fang B. Gannon P. Ding P. Li X.. (2010). *Arabidopsis* snc2-1D activates receptor-like protein-mediated immunity transduced through WRKY70. Plant Cell 22, 3153–3163. 10.1105/tpc.110.074120 20841424 PMC2965549

