# Supplementary figures and images for: Identification of receptor-like proteins induced by Sclerotinia sclerotiorum in Brassica napus
Source: Front Plant Sci. 2022 Aug 16;13:944763. doi: 10.3389/fpls.2022.944763 (PMC9429810; doi:10.3389/fpls.2022.944763)

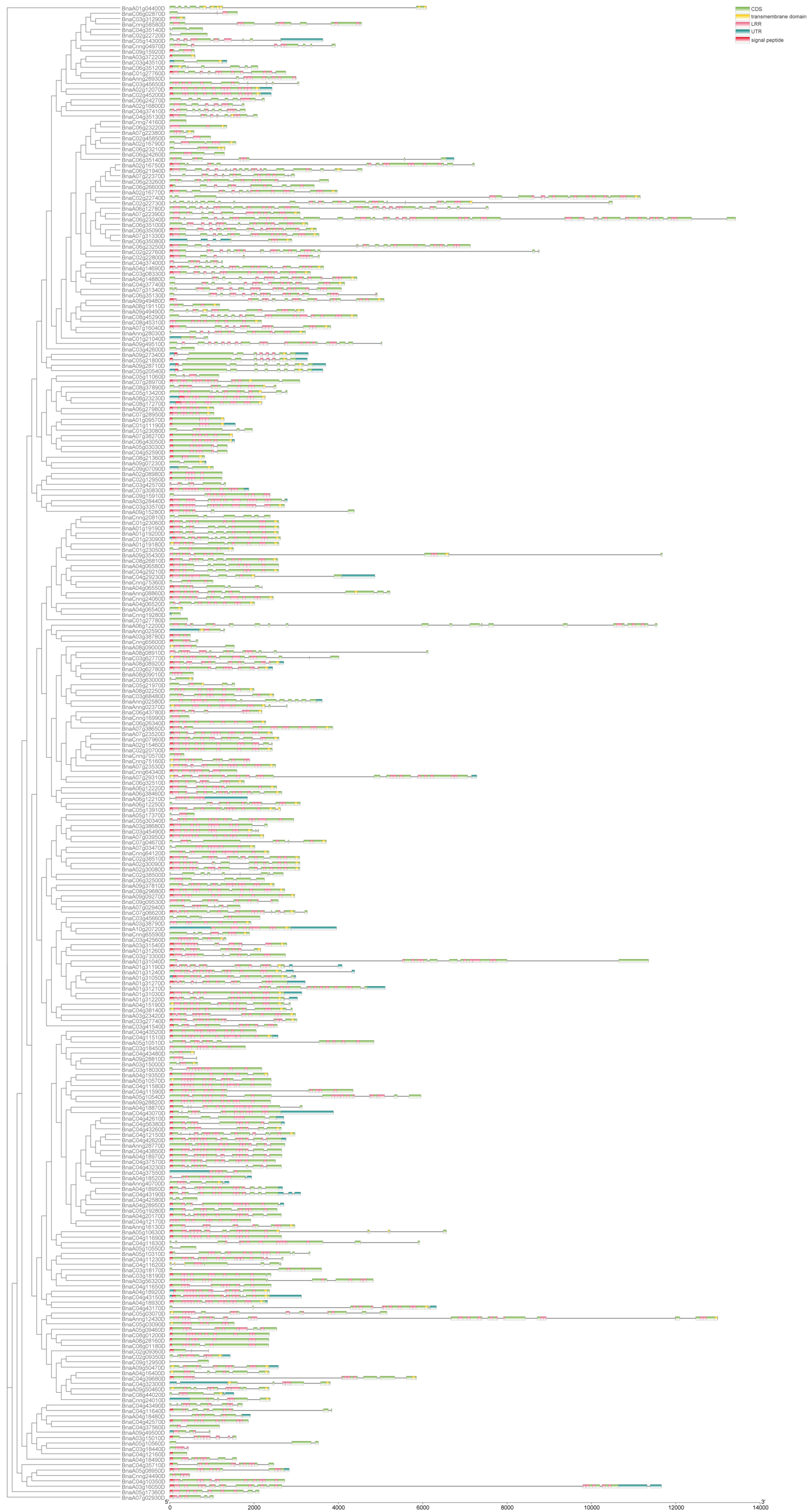

Supplement: Supplementary Figure 1 — Gene and protein structures of RLP genes in B. napus. The green bar represents CDS, the black line represents intron, and the blue bar represents UTR. The pink bar stands for the LRR motif, the yellow bar for the transmembrane domain, and the red for the signal peptide. [file Image_1.JPEG]

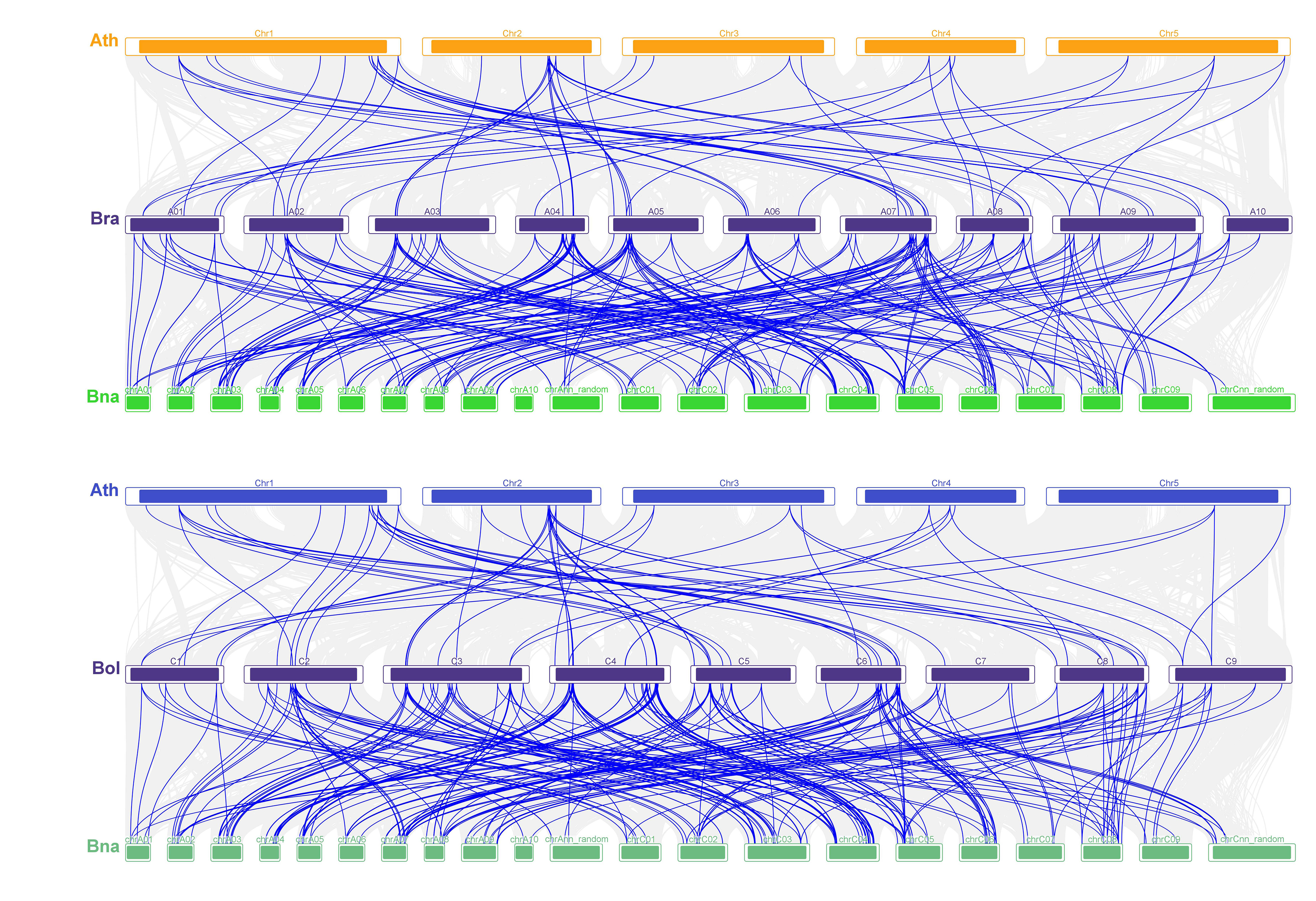

Supplement: Supplementary Figure 4 — Syntenic relationship of RLP-encoding genes between A. thaliana and Brassicaceae species. [file Image_4.JPEG]

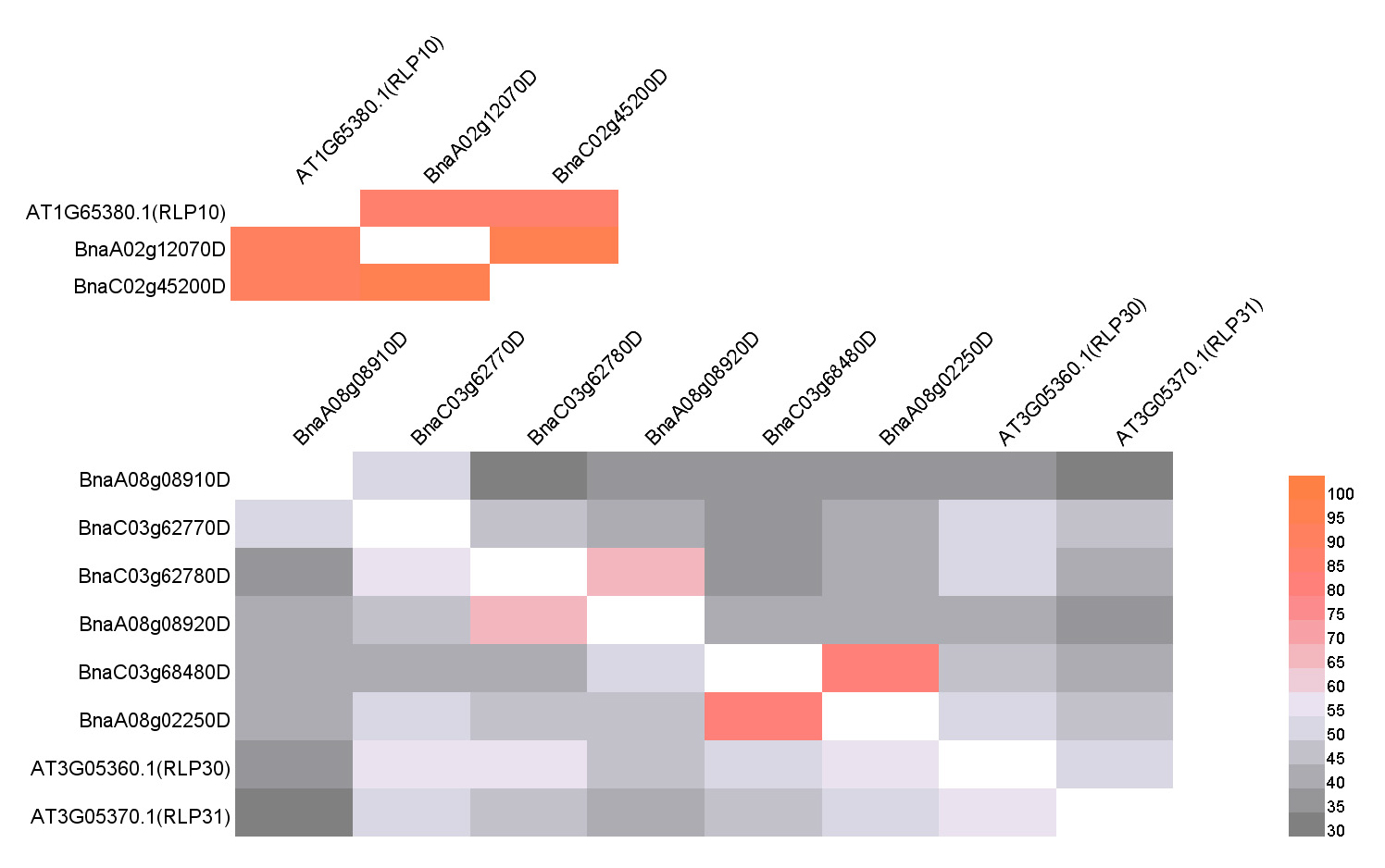

Supplement: Supplementary Figure 7 — Protein similarity and identity of RLP families. Data on the upper right present the protein sequence identity, and data on the bottom left present the protein sequence similarity. [file Image_7.JPEG]
